# The Persian version of the body esteem scale among Iranian adolescents: a translation, psychometrics, and network analysis

**DOI:** 10.3389/fpsyg.2024.1296498

**Published:** 2024-01-29

**Authors:** Hamid Sharif-Nia, Erika Sivarajan Froelicher, Long She, Azar Jafari-Koulaee, Sima Hejazi, Hasan Mosazadeh, Amir Hossein Goudarzian, Ghaem Hasan Nejad

**Affiliations:** ^1^Psychosomatic Research Center, Mazandaran University of Medical Sciences, Sari, Iran; ^2^Department of Nursing, Amol Faculty of Nursing and Midwifery, Mazandaran University of Medical Sciences, Sari, Iran; ^3^Department of Physiological Nursing, School of Nursing, University of California, San Francisco, San Francisco, CA, United States; ^4^Department of Epidemiology and Biostatistics, School of Medicine, University of California, San Francisco, San Francisco, CA, United States; ^5^Sunway Business School, Sunway University, Sunway, Malaysia; ^6^Faculty of Medical Sciences, Tarbiat Modares University, Tehran, Iran; ^7^Bojnurd Faculty of Nursing, North Khorasan University of Medical Sciences, Bojnurd, Iran; ^8^Department of Psychology, Kazimierz Wielki University of Bydgoszcz, Bydgoszcz, Poland; ^9^Student Research Committee, Mazandaran University of Medical Sciences, Bojnourd, Iran; ^10^Student Research Committee, Tehran University of Medical Sciences, Bojnourd, Iran

**Keywords:** body esteem, validity, reliability, psychometrics, Iran, network analysis

## Abstract

**Introduction:**

The psychometric properties of the body esteem scale have not been assessed in Iran. Therefore, the aim of this study was to translate and determine the validity and reliability of the Persian version of the body esteem scale among Iranian adolescents.

**Methods:**

The sample of this methodological study consisted of 504 adolescents [mean age: 16.55 (SD = 1.54) years] living in Tehran City, Iran. After translation of the scale, its content validity (quantitative and qualitative) and structural (exploratory and confirmatory factor analysis), convergent, and discriminant validity were evaluated. Exploratory graph analysis was performed to determine the number of factors. Cronbach’s alpha, composite reliability, and maximal reliability were calculated.

**Results:**

In the content validity evaluation step, all items had acceptable scores and were retained. The results of exploratory factor analysis with Promax rotation and exploratory graph analysis extracted three factors accounting for 49.49% of the variance, comprising 18 items. Furthermore, after necessary modifications during CFA, the final model was approved. Convergent and discriminant validity were confirmed. Cronbach’s alpha, CR, and MaxR for all constructs were greater than 0.7, demonstrating good internal consistency and construct reliability.

**Conclusion:**

According to the results, the Persian version of the body esteem scale has a valid structure and acceptable reliability. Health professionals, in many ways, can use this scale.

## Introduction

Body esteem refers to the self-evaluation of one’s body or appearance, which is conceptualized as a global construct (Mendelson et al., 2000). Dissatisfaction with the body or appearance and concerns related to it have been reported in different age groups, and the concept of body esteem has attracted the attention of researchers (Adams et al., 2005; Frost et al., 2018; Olchowska-Kotala, 2018). Among different age groups, adolescents experience a vulnerable period with rapid physical and cognitive changes during puberty that can cause lower levels of body esteem (Arim et al., 2006; Voelker et al., 2015). Puberty can have serious consequences for body esteem. The maturational deviance hypothesis states that deviance from the norm puts adolescents at risk of developing body concerns (Frisén et al., 2015).

In addition, adolescents are under pressure from social factors such as their peers and their living environment, psychological factors such as personality characteristics and self-esteem, and cultural factors to achieve aesthetic ideals (Mellor et al., 2009; Sheldon, 2010). Researchers indicated that boys and girls with early and late maturing tend to be those who report a lower level of body esteem (Frisén et al., 2015). Low body esteem is one of the factors that can disturb the internal balance of the body, which is created by meeting physical, psychological, and social needs and can also cause social, psychological, and physiological problems (Davis and Katzman, 1997; Heidari and Ghodusi, 2015). Studies have shown that individuals with a lower level of body esteem were more likely to experience anabolic steroid abuse (Parent, 2013), eating disorders (Rayner et al., 2013), and social anxiety (Strelan and Hargreaves, 2005). Body esteem was supported as a mechanism that influenced the level of depression in adolescent girls (Hamlat et al., 2015). Body esteem can affect physical nature, identity formation, self-esteem, hope, mental health, and ultimately success in various aspects of life (Van den Berg et al., 2007; Heidari and Ghodusi, 2015). Therefore, accurate understanding and evaluation of body esteem are very important, especially in adolescents.

There are two widely used scales for measuring body esteem that have been presented by Franzoi and Shields (1984) and Mendelson et al. (2001), respectively. The body esteem scale developed by Mendelson is a widely used self-report scale for body esteem. This scale is adapted for use in adolescents and adults and is more concise and accurate. The original version of this scale includes three subscales: appearance (evaluation of general feelings and satisfaction with overall appearance), weight (evaluation of general feelings and satisfaction with weight), and attributes (evaluations attributed to others about an individual’s appearance) (Mendelson et al., 2001). The wide conceptualization of this scale of appearance and the evaluation of the cognitive and emotional image of the body make this scale valuable. Furthermore, this scale does not focus exclusively on weight and shape, but the Mendelson scale comprehensively considers all concerns related to body esteem (Smith et al., 2022). The body esteem scale developed by Mendelson has been psychometrically tested in several populations and cultures. It has also been translated into and validated in Spanish (Beltrán-Garrayo et al., 2022), Italian (Confalonieri et al., 2008), and Turkish (Arslan et al., 2020).

The Iranian culture, rooted in a rich history and shaped by a blend of tradition and contemporary influences, plays a pivotal role in shaping the body esteem and psychological characteristics of adolescents. With a strong emphasis on collectivism, modesty, and traditional gender roles, Iranian youth often navigate a complex interplay between cultural expectations and globalized ideals (Shoraka et al., 2019). Religious influences, particularly from Islam, contribute to shaping perceptions of beauty and self-worth. Moreover, the impact of media, educational systems, and family dynamics further weaves into the intricate fabric of adolescent development in Iran (Shoraka et al., 2019). Understanding these cultural nuances is crucial for exploring the intricate relationship between cultural context and the psychological well-being of Iranian adolescents. Regarding this issue, we should culturally adapt this scale for use by the Iranian population.

Thus far, the validity and reliability of the Persian version of the body esteem scale have not been assessed in Iran. Therefore, this study was conducted with the aim of translating and determining the reliability and validity of the Persian version of the body esteem scale (BES-P) in Iranian adolescents through psychometric properties and network analysis.

## Methods

### Design

This methodological study was carried out in 2023. The study consisted of two phases: (1) translation of the scale and (2) psychometric evaluation of the translated scale.

### Participants

Adolescents living in Tehran (Tehran, Iran) who fulfilled the following criteria were enrolled in this study via the convenience sampling method: (i) reading proficiency in Farsi; (ii) absence of advanced psychological disorders such as major depression or schizophrenia (assessed by self-expression); (iii) age between 12 and 18; and (vi) access to the internet and social media. Adolescents with major depressive disorders were not included in the study because of the high likelihood that depression would have an effect on body image.

MacCallum et al. (1999) recommended a sample size of at least 200 cases for psychometric studies (MacCallum et al., 1999). Therefore, we decided to extend an invitation to 504 people due to the necessity of two different samples for structural validity.

The scale URL was sent to parents through social networks. In the beginning, there was an online parental consent form for the participation of adolescents in the research, which was filled out by parents. Then, parents would give the questionnaire to the adolescent to complete. The participants were given a thorough explanation of the study’s goals and methods, as well as assurances that their participation was entirely voluntary.

The Ethics Committee of Mazandaran University of Medical Sciences (Sari, Iran) gave its approval to this study (Ethics code: IR.MAZUMS.REC.1402.432).

### Original version of scale

The original version of BES was conceptualized and developed by Mendelson et al. (2001). This scale was developed to assess the components of body esteem. It has 23 items with a Likert scale scoring based on (0 = never to 4 = always) (Mendelson et al., 2001). Furthermore, it should be declared that the original version of this scale was designed for adults and adolescents.

### Phases of the study

#### Translation

To conduct this study, we secured written permission from the scale’s developer to use the BES. Subsequently, the scale was translated from English to Persian following the Gudmundsson (2009) translation protocol. Two proficient English-Persian translators independently translated the BES into Persian. An expert panel, comprising some of the authors of this article and two professional translators, meticulously reviewed and amalgamated the two translations to create a Persian version of the BES. Subsequently, a Persian-English translator was engaged to translate the BES-P back into English. The panel of experts reviewed and approved this final version.

#### Psychometric evaluation

##### Content validity assessment

###### Qualitative content validity assessment

Five experts were given the BES-P in order to evaluate and offer comments on the wording, item placement, and item scaling.

###### Quantitative content validity

Five experts who worked on the content validity rated the essentiality of the BES-P items on a 3-point response scale (not essential, useful but not necessary, and essential) (Hosseini et al., 2022). A modified kappa statistic was then established. Accounting for chance agreement helped to reveal the level of agreement among the experts. The following formula was used to calculate the kappa (K) by substituting the content validity index (I-CVI) and probability of chance agreement (Pc):


$$K=\left(I-CVI-P_{C}\right)/\left(1-P_{C}\right)$$

The probability of concordance is usually 0.5, and the power for the number of experts is Pc = (0.5) n. A value for the kappa statistic of more than 74% is regarded as excellent, between 60 and 73.99% as good, and between 40 and 59% as showing unacceptable agreement between the experts. Each item on the scale must have a content validity index of at least 78%, according to Polit and Beck (2010), in order for an item to be deemed excellent. Moreover, when the number of expert panel members increases, the probability of coincidence decreases, and the content validity values and kappa statistics for each item converge.

The expert panel members are asked to assess the scales’ comprehensiveness as the last step of the content validity evaluation. In order to determine whether the items and any of the scale’s dimensions are complete and comprehensive, samples of content for the concept dimensions and operational definitions are evaluated. Additionally, reviewers should decide whether a particular item should be added or removed. For each of the dimensions and for the entire scale’s comprehensiveness, the proportions of agreement are calculated based on their assessment. The number of experts who said the scale was comprehensive is divided by the total number of experts on the panel to arrive at this result.

##### Structural validity

To test the structural validity, the original dataset (*n* = 504) was randomly divided into two datasets with 252 cases each. With the first random dataset (*n* = 252), maximum likelihood exploratory factor analysis (MLEFA) with Promax with Kaiser normalization rotation and exploratory graph analysis methods were conducted to determine the factor structure. In the field of network psychometrics, exploratory graph analysis (EGA) is a new method that determines the number of factors underlying multivariate data. EGA generates a network plot, which is a visual guide that displays how many factors should be kept, which items cluster together, and how strongly they are related (Golino and Epskamp, 2017). In the present study, EGA was performed by JASP0.18.0.0 software. The Kaiser–Meyer–Olkin (KMO) > 0.8 and Bartlett’s test of sphericity to be significant (*p* < 0.01) were referred to ensure the data was relevant and appropriate for performing the factor analysis. Eigenvalues of more than 1, communalities of more than 0.2, and factor loadings of more than 0.5 with scree plots were also used for the factor extraction (Cattell, 1966; Field, 2013; Sharif Nia et al., 2021b). The MLEFA was performed using SPSS version 27.

In the next step, the factor structure obtained from MLEFA was analyzed and confirmed by conducting confirmatory factor analysis (CFA) based on the second random dataset (*n* = 252) using AMOS version 27. The following model fit indices were used to assess the model fit: comparative fit index (CFI), normed fit index (NFI), goodness of fit index (GFI), relative fit index (RFI), and incremental fit index (IFI) were > 0.9; that of root mean square error of approximation (RMSEA) was <0.08; and for minimum discrepancy function divided by degrees of freedom (CMIN/DF), < 3 was considered good (Hosseini et al., 2022).

### Normal distribution, outliers, and missing data

Skewness (±3) and kurtosis (±7) were used to individually investigate the univariate distribution of the data. Furthermore, multivariate normality distribution was assessed by the Mardia coefficient of multivariate kurtosis (<8). Mahalanobis d-squared (*p* < 0.001) was used to determine whether there were any multivariate outliers (Sharif Nia et al., 2021a). The missing data were assessed using multiple imputations, and the average participant response was used to replace the missing data (Patrician, 2002).

#### Convergent and discriminant validity

For convergent validity, composite reliability (CR) should be greater than 0.7, and average variance extracted (AVE) should be greater than 0.5 for each construct. Fornell and Larcker (1981) stated that for psychological constructs, if AVE is less than 0.5 but CR is more than 0.6, the convergent validity can be considered acceptable.

With respect to discriminant validity, this study used the heterotrait–monotrait ratio (HTMT) of the correlations criterion, where the HTMT ratio between all constructs should be less than 0.85 to achieve discriminant validity (Henseler et al., 2015).

#### Reliability

Cronbach’s alpha, McDonald’s omega coefficient, composite reliability (CR), and maximal reliability (MaxR) were calculated to gauge the internal consistency and construct reliability (Javali et al., 2011; She et al., 2021). If the scale’s Cronbach alpha was more than 0.7 and CR and MaxR were more than 0.7, it was deemed to have good internal consistency and construct reliability (Mayers, 2013).

##### Body esteem score

Descriptive statistics were employed to calculate the mean score of body esteem. Additionally, an independent samples t-test was conducted to evaluate differences between the groups of men and women with respect to body esteem.

## Results

### Demographic characters

The mean age of the participants was 16.55 (SD = 1.54) years. Among the participants, 112 (22.2%) were women, and 392 (77.8%) were men. The history of cosmetic surgery in the family of the participants was 36.5% (*n* = 184). On the other hand, 94 participants (18.7%) reported that they had friends who were not satisfied with their appearance. Furthermore, 32 adolescents (6.3%) included in the study were not satisfied with their sex.

### Content validity

The reviewers qualitatively examined the content’s validity following the instrument’s revision. All scale items should be present because the CVR value was higher than 4.9 for every item. All items on the scale had a sufficient relationship with the instrument’s concept when taking into account the obtained scores (CVI and K coefficients of the items). Using the mean method (S-CVI/Ave), the content validity index was determined for the entire instrument to be 0.91. Additionally, the S-CVI/UA universal consensus method yielded an exponent of 0.79. Finally, the content validity of each item was confirmed.

### Structural validity

**Figure 1:**
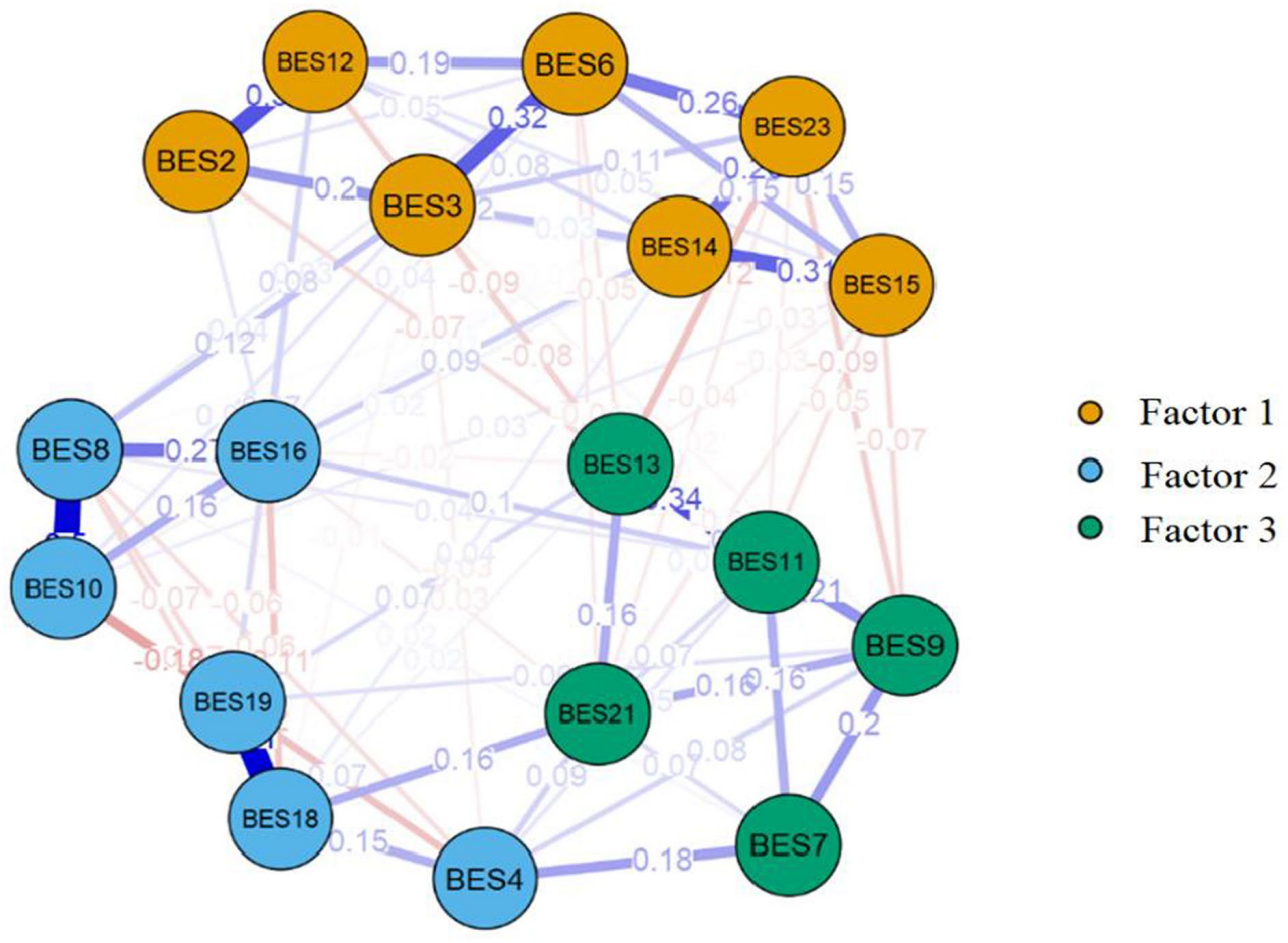
Exploratory graph analysis.

The results of MLEFA with Promax with Kaiser Normalization rotation using the first random dataset (*n* = 252) extracted three factors accounting for 49.49% of the variance, comprising 18 items. Items 1, 5, 17, 20, and 22 were removed from the original version due to communalities of less than 0.2 and factor loadings of less than 0.5. Moreover, the results of the KMO (0.920) and Bartlett’s test of sphericity (*p* < 0.001, 2687.564, *df* = 153) showed that the sampling is adequate and appropriate for conducting factor analysis. The detailed results of the MLEFA are shown in Table 1. Furthermore, the EGA is shown in Figure 1.

**Table 1:** Result of MLEFA on the three factors Persian version of body esteem scale (*N* = 252).

Factor	Items	Factor loading	h^2^	λ	% variance
Weight	Q_4_. I am preoccupied with trying to change my body weight	0.506	0.492	3.485	19.36%
	Q_8_. I am satisfied with my weight	0.898	0.788		
	Q_10_. I really like what I weigh	0.871	0.750		
	Q_16_. I feel I weigh the right amount for my height	0.687	0.596		
	Q_18_. Weighing myself depresses me	0.757	0.611		
	Q_19_. My weight makes me unhappy	0.796	0.670		
Appearance satisfaction	Q_2_. Other people consider me good-looking	0.680	0.445	3.463	19.23%
	Q_3_. I’m proud of my body	0.599	0.616		
	Q_6_. I like what I see when I look in the mirror	0.764	0.664		
	Q_12_. People my own age like my looks	0.711	0.428		
	Q_14_. I’m as nice-looking as most people	0.734	0.491		
	Q_15_. I’m pretty happy about the way I look	0.709	0.562		
	Q_23_. I look as nice as I’d like to	0.715	0.647		
Appearance anxiety	Q_7_. There are lots of things I’d change about my looks if I could.	0.619	0.331	1.962	10.90%
	Q_9_. I wish I looked better	0.626	0.557		
	Q_11_. I wish I looked like someone else	0.804	0.581		
	Q_13_. My looks upset me	0.513	0.630		
	Q_21_. I worry about the way I look	0.524	0.489		

h^2^, communalities; λ, Eigenvalues.

**Figure 2:**
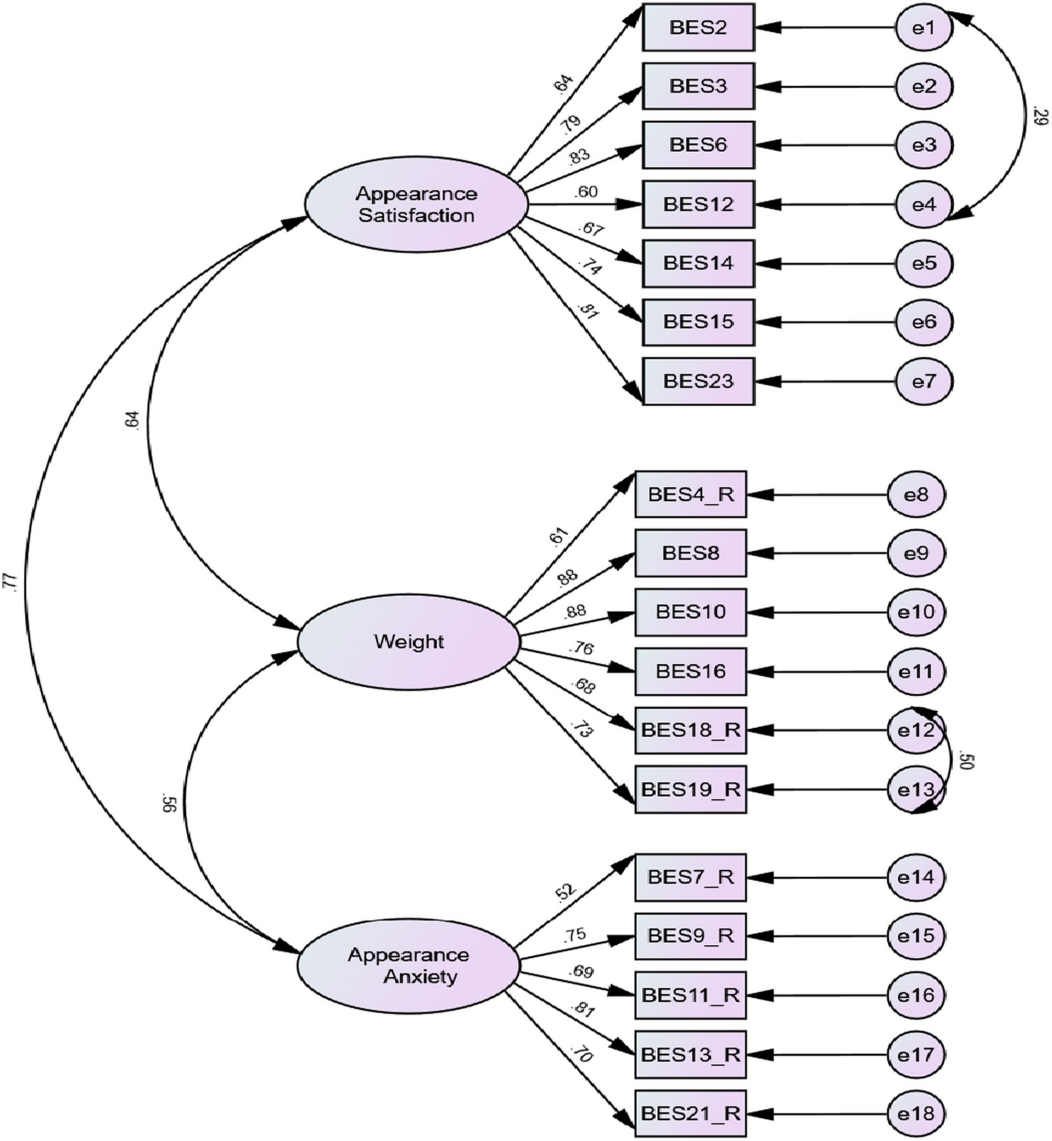
Results of the CFA and factor loadings.

The CFA was conducted to confirm and validate the factor structure obtained from MLEFA using the second random dataset (*n* = 252). The initial results showed that the data did not fit the model well, as evidenced by [*χ*^2^(132) = 418.505, *p* < 0.001, *χ*^2^/*df* = 3.170, CFI = 0.890, IFI = 0.891, TLI = 0.873, SRMR = 0.067, RMSEA (90% C.I.): 0.093 [0.083, 0.103]]. Referring to the results of the modification indices, two pairs of measurement errors (between items 2 and 12 and items 18 and 19) were allowed to freely covary to improve the model fit. Figure 2 shows the revised measurement mode with all factor loadings greater than 0.5 (ranging from 0.517 to 0.883). The revised model showed a good fit, as evidenced by goodness-of-fit indices [*χ*^2^(130) = 339.907, *p* < 0.001, *χ*^2^/*df* = 2.594, CFI = 0.920, IFI = 0.920, TLI = 0.905, SRMR = 0.066, RMSEA (90% C.I.): 0.080 [0.070, 0.091]].

### Convergent and discriminant validity

Table 2 shows the results of the CFA. The results showed that AVE for factor appearance satisfaction and weight were greater than 0.5, indicating good convergent validity. The AVE for appearance anxiety was less than 0.5 but close to 0.5 (0.487). Hence, with the factor of appearance anxiety’s CR greater than 0.7, it can be concluded that convergent validity for all constructs has been established. As for discriminant validity, the results of the HTMT ratio showed that the correlation between appearance satisfaction and weight (0.626), between appearance satisfaction and appearance anxiety (0.717), and between weight and appearance anxiety (0.615) was lower than 0.85, demonstrating good discriminant validity for all constructs.

**Table 2:** Results of the convergent validity and construct reliability (*n* = 252).

Factors	α	Ω	CR	MaxR	AVE
Weight	0.897	0.885	0.899	0.914	0.600
Appearance satisfaction	0.891	0.892	0.891	0.901	0.540
Appearance anxiety	0.823	0.826	0.823	0.843	0.487

α, Cronbach’s alpha; Ω, McDonald’s omega.

### Reliability

As for construct reliability, Cronbach’s alpha, CR, and MaxR for all constructs were greater than 0.7, demonstrating good internal consistency and construct reliability. Furthermore, based on Table 2, McDonald’s omega of all of the latent variables was in an acceptable range.

#### Body esteem score

In the overall population, the mean score for body esteem was 73.10 (SD = ±7.29, 95%CI = 72.19, 74). Furthermore, there were no significant differences (*p* = 0.67) in body esteem scores between men (73.87, SD = ±7.14) and women (70.39, SD = ±7.20).

## Discussion

The main objective of this study was to translate the body esteem scale into the Persian language and examine its reliability and validity among Iranian adolescents. By testing it on a group of Iranian adolescents aged 12–18, the body esteem scale displayed a satisfactory factor structure, validity, and reliability.

In this study, the structural validity of the body esteem scale was investigated, and it was found that items loaded on the first to third factor explained 42.20% (7 items), 9.09% (6 items), and 6.20% (5 items) of the total variance, respectively. The combined variance explained by these three factors was 57.49%. Overall, these findings support the validity of the body esteem scale as a measure of body esteem among 12–18-year-old Iranian adolescents. The body esteem scale developed by Mendelson has been psychometrically tested in several populations and cultures, including Spanish (Beltrán-Garrayo et al., 2022), Italian (Confalonieri et al., 2008), and Turkish adolescents (Arslan et al., 2020), and its validity has been confirmed. In the Spanish version, like in the present study, three factors were extracted with 62.44% of the total variance (Beltrán-Garrayo et al., 2022). Furthermore, in line with the present study, three components were extracted with 57% of the variance from the Italian version (Confalonieri et al., 2008). The Turkish version explored three factors that explained 58.98% of the total variance (Arslan et al., 2020).

The first factor is called appearance satisfaction. Appearance satisfaction and body esteem during adolescence can have a significant impact on a young person’s overall well-being and self-esteem. This period of development is characterized by physical changes, social pressures, and heightened self-consciousness, which can influence how adolescents perceive and evaluate their bodies (Yang et al., 2020). Furthermore, the second factor explained weight. The relationship between weight and body esteem in adolescence refers to how an adolescent’s perception of their body weight can impact their overall satisfaction and self-evaluation of their physical appearance. Adolescents who feel satisfied with their weight are more likely to have positive body esteem, while those who feel dissatisfied with their weight may experience negative body esteem. It is important to note that this relationship is complex and can be influenced by various factors, including societal standards of beauty, cultural norms, peer comparisons, media influences, and personal experiences (Yang et al., 2020). Weight-related concerns and body esteem in adolescence can play a role in the development of body image dissatisfaction, eating disorders, and psychological well-being. Encouraging a healthy body image, promoting body diversity, and emphasizing self-acceptance can help support positive body esteem among adolescents, regardless of weight. The third factor is called appearance anxiety. Appearance anxiety, also known as appearance-related anxiety or body image anxiety, is characterized by excessive worry, self-consciousness, and fear about one’s physical appearance, including body shape, weight, facial features, and other aesthetic aspects. Adolescents who experience higher levels of appearance anxiety may engage in frequent self-scrutiny, comparison with peers, and fear of negative evaluation from others (Perez et al., 2023). Appearance anxiety can have a negative impact on body esteem in adolescence. When adolescents are highly anxious about their appearance, they may develop a negative body image and experience dissatisfaction with their physical attributes. This can lead to lowered self-esteem, reduced self-confidence, and a diminished sense of overall well-being (Yang et al., 2020; Perez et al., 2023).

The results of the confirmatory factor analysis suggest that the hypothetical model fits well with the data, providing support for the three-factor model. All three factors were found to be strongly correlated with the total score of body esteem.

In the present study, the internal consistency of the extracted factors of the body esteem scale was between 0.823 and 0.897. In fact, it indicates that the items of the questionnaire are measuring a similar concept, and the items on the scale are accurate, reliable, repeatable, and desirable. The reliability of the Spanish (Beltrán-Garrayo et al., 2022), Italian (Confalonieri et al., 2008), and Turkish (Arslan et al., 2020) translations of this scale was also proven to be adequate. The results of all the studies identified are consistent with the findings of this study. This scale can be used by clinicians for early identification of body esteem problems, allowing for interventions to start before the problem becomes severe and irreversible.

Another main result of the present study was the score of body esteem in our population and the similarity of this variable in the men and women’s populations. Some previous studies concluded that body esteem is different between men and women (Kaminski and Hayslip, 2006; Grossbard et al., 2009). The composition of the study sample can greatly influence the outcomes. If the sample consists of individuals who have similar characteristics, such as age, cultural background, or socioeconomic status, this homogeneity could lead to a lack of significant differences in body esteem scores. It is important to ensure that the sample is diverse and representative of the population being studied. Cultural and societal shifts over time can impact body esteem. In some regions and societies, traditional gender roles and expectations may be changing, leading to more similar body esteem experiences between men and women. For example, increased emphasis on promoting body positivity and acceptance may contribute to reduced gender differences in body esteem. Respondents might provide socially desirable answers, especially in surveys about body esteem, which can mask true differences. This is more likely when participants feel self-conscious about their responses and want to conform to societal expectations. Ensuring anonymity and privacy in data collection can help mitigate this bias.

### Limitations and strengths

One constraint of the current investigation was the potential limitations of applying its findings to a broader population. Since the study was carried out exclusively in the Tehran province and body image perceptions can be affected by social norms and cultural values, it is important to exercise caution when extending these conclusions to the wider public. Furthermore, one limitation is the lack of random sampling, limiting the generalizability of our findings. Nevertheless, our study has numerous strengths. The use of exploratory graph analysis to identify factors was one of the study’s strengths. Another advantage of this study is the calculation of the Omega-McDonald’s coefficient in addition to Cronbach’s alpha.

### Implications

Applying the body esteem scale to Iranian adolescents acknowledges the importance of considering cultural differences. This can lead to a better understanding of how body image is shaped by Iranian cultural norms, values, and beauty ideals. Applying the scale can provide evidence of the role media, peer pressure, and cultural norms play in shaping body image perceptions among Iranian adolescents. This information can guide discussions about the potential impact of these influences on self-esteem. The results obtained from the body esteem scale can contribute to raising public awareness about the significance of positive body image and self-esteem in Iranian society. This, in turn, can stimulate discussions about promoting healthier body image ideals. The scale’s application might lead to the development of culturally adapted versions that are more relevant to the Iranian context. This can enhance the accuracy of measuring body esteem in this specific population.

## Conclusion

To the best of our knowledge, this is the first study to assess the validity and reliability of the body esteem scale in Iranian 12 to 18-year-old adolescents, covering several aspects related to body esteem, including appearance satisfaction, weight, and appearance anxiety. According to the results, the Persian version of the body esteem scale has a valid structure and acceptable reliability. This scale can be used by health professionals in many ways.

## Data availability statement

The raw data supporting the conclusions of this article will be made available by the authors, without undue reservation.

## Ethics statement

The studies involving humans were approved by Mazandaran University of Medical Sciences (Sari, Iran) (Ethics code: IR.MAZUMS.REC.1402.432). The studies were conducted in accordance with the local legislation and institutional requirements. Written informed consent for participation in this study was provided by the participants’ legal guardians/next of kin.

## Author contributions

HS-N: Conceptualization, Formal analysis, Methodology, Supervision, Writing – review & editing. ES: Writing – original draft, Writing – review & editing. LS: Formal analysis, Writing – review & editing. AJ-K: Data curation, Writing – original draft, Writing – review & editing. SH: Writing – original draft, Writing – review & editing. HM: Writing – original draft, Writing – review & editing. AG: Conceptualization, Writing – original draft, Writing – review & editing. GH: Data curation, Writing – review & editing.
